# Effectiveness Comparisons of Drug Therapy on Chronic Subdural Hematoma Recurrence: A Bayesian Network Meta-Analysis and Systematic Review

**DOI:** 10.3389/fphar.2022.845386

**Published:** 2022-03-17

**Authors:** Wanli Yu, Weifu Chen, Yongxiang Jiang, Mincai Ma, Wei Zhang, Xiaolin Zhang, Yuan Cheng

**Affiliations:** Department of Neurosurgery, The Second Affiliated Hospital of Chongqing Medical University, Chongqing, China

**Keywords:** chronic subdural hematoma, recurrence, drug therapy, effectiveness, Bayesian network chronic subdural hematoma, Bayesian network meta-analysis

## Abstract

**Objectives:** We aim to compare the effectiveness of different drug treatments in improving recurrence in patients with chronic subdural hematoma (CSDH).

**Methods:** Eligible randomized controlled trials (RCTs) and prospective trials were searched in PubMed, Cochrane Library, and Embase, from database inception to December 2021. After the available studies following inclusion and exclusion criteria were screened, the main outcome measures were strictly extracted. Taking the random-effects model, dichotomous data were determined and extracted by odds ratio (OR) with 95% credible interval (CrI), and a surface under the cumulative ranking curve (SUCRA) was generated to calculate the ranking probability of comparative effectiveness among each drug intervention. Moreover, we used the node-splitting model to evaluate inconsistency between direct and indirect comparisons of our network meta-analysis (NMA). Funnel plots were used to evaluate publication bias.

**Results:** From the 318 articles found during initial citation screening, 11 RCTs and 3 prospective trials (*n* = 3,456 participants) were ultimately included in our study. Our NMA results illustrated that atorvastatin + dexamethasone (ATO+DXM) (OR = 0.06, 95% CrI 0.01, 0.89) was the most effective intervention to improve recurrence in patients with CSDH (SUCRA = 89.40%, 95% CrI 0.29, 1.00). Four drug interventions [ATO+DXM (OR = 0.06, 95% CrI 0.01, 0.89), DXM (OR = 0.18, 95% CrI 0.07, 0.41), tranexamic acid (TXA) (OR = 0.26, 95% CrI 0.07, 0.41), and ATO (OR = 0.41, 95% CrI 0.12, 0.90)] achieved statistical significance in improving recurrence in CSDH patients compared with the placebo (PLB) or standard neurosurgical treatment (SNT) group.

**Conclusion:** Our NMA showed that ATO+DXM, DXM, ATO, and TXA had definite efficacy in improving recurrence in CSDH patients. Among them, ATO+DXM is the best intervention for improving recurrence in patients with CSDH in this particular population. Multicenter rigorous designed prospective randomized trials are still needed to evaluate the role of various drug interventions in improving neurological function or outcome.

**Systematic Review Registration:** (https://www.crd.york.ac.uk/prospero/display_record.php?RecordID=299491), identifier (CRD 42022299491).

## Introduction

Chronic subdural hematoma (CSDH) is a common neurologic disorder that mainly affects the elderly, and the morbidity of CSDH has been reported to be up to 20.6 per 100,000 persons per year (Yang and Huang, 2017). Besides the fact that the population of patients with CSDH is still growing in an aging society (Balser et al., 2015; Rauhala et al., 2019), slow bleeding from vascular injury after head trauma has been considered as the main cause of CSDH development, and treatment with burr-hole craniotomy often improves patients’ symptoms definitely, but a prominent problem is that CSDH often recurs (33%) and always results in poor outcomes (Zhang, 2021). However, the pathogenesis involved in the recurrence of CSDH remains unclear. Some literature indicated that the recurrence formation of CSDH mainly includes the theories of inflammation (Frati et al., 2004), angiogenesis (Hohenstein et al., 2005), exudates (Thomas et al., 2019), recurrent microbleeds, and local coagulopathy (Holl et al., 2018). Therefore, many related drug therapies that could help resolve the recurrence of CSDH have emerged, thus avoiding multiple repeat surgeries for CSDH. Steroids may bring antiangiogenic and anti-inflammatory effects (Kalamatianos et al., 2013), tranexamic acid (TXA) may exert an antifibrinolytic effect (de Faria et al., 2021), and goreisan (GRS) acts on aquaporin to regulate water permeability, so it can inhibit the formation and growth of CSDH theoretically. In the case of understanding the underlying pathophysiological processes described above, many previous clinical trials of drugs have been performed on CSDH patients after surgery to improve the recurrence, but few drugs have been rigorously compared and ranked according to their effectiveness in a study, and optimal drug interventions are still being debated (Holl et al., 2018). Accordingly, we had analyzed the existing evidence and presented a first Bayesian network meta-analysis (NMA) to identify the most effective drug intervention that could improve the recurrence in patients with CSDH from a macroscopic aspect by comparing multiple treatments simultaneously.

## Materials and Methods

This study followed guidance and reports for systematic reviews in the Preferred Reporting Items for Systematic Reviews and Meta-Analyses (PRISMA) NMA checklist (Hutton et al., 2015) and the Cochrane Handbook (Cochrane Handbook for Systematic Reviews of Interventions, 2011). All the analyses were based on the previously published research; therefore, they do not require ethical approval and patient consent.

### Literature Search

Extensive preliminary literature retrieval was done by searching PubMed, Embase, and Cochrane Central Register of Controlled Trials without restriction by year and language, to identify all relevant prospective studies and randomized controlled trials (RCTs) from their inception to December 1, 2021. The Medical Subject Headings (MeSH) and text terms were combined with Boolean logical operators using “Chronic subdural hematoma,” “Tranexamic acid,” “Dexamethasone,” “Atorvastatin,” “Goreisan,” “Celecoxib,” “Antithrombotic,” “Prospective cohort studies,” “Randomized controlled trials,” and other relevant conceptual keywords. The detailed search strategies and links of the final search citations are summarized in the Supplementary Material search strategies.

### Selection Criteria

The whole eligible citations were evaluated, the title and abstract of the citations gained from the search were filtered, and citations that failed to meet the inclusion criteria or were repeatedly published were excluded. The full text was read carefully to further assess the articles’ relevance according to the inclusion criteria. Additionally, the references in the included articles were evaluated to further explore the relevant research. All citations were downloaded and regulated in Endnote X9 (Thompson ISI Research Soft, Philadelphia, PA, USA).

### Inclusion and Exclusion Criteria

The inclusion criteria were set as follows: 1) all included patients were clearly diagnosed with CSDH; 2) comparative studies include RCTs or prospective studies; 3) each trial should include at least 20 patients; 4) main outcome measures are clearly reported.

The exclusion criteria are as follows: 1) recurrent CSDH and 2) patients <18 years old.

The main outcome measurements were set as recurrence rates in patients with CSDH. Recurrence was defined as the occurrence of symptomatic CSDH that required reoperation or needed a new intervention during the study period.

### Data Abstraction and Quality Appraisal

Two authors (WL-Y and XL-Z) independently extracted and summarized the data that met the inclusion and exclusion criteria. The demographic characteristics and geographic data of all included articles were first analyzed against a pre-customized outcome data collection table. Study name, first author, publication year, country and region, basic characteristics, and other relevant data were extracted as baseline data.

The Cochrane Bias Risk Tool was used to evaluate the risk of bias (ROB) in the included studies using the software Review Manager (Version 5.4) (Cochrane Handbook for Systematic Reviews of Interventions, 2011). The Cochrane bias risk criteria included the following six components: selection bias, performance bias, detection bias, attrition bias, reporting bias, and other sources of bias.

### Statistical Analyses

Minimally informative prior distributions of the Bayesian random-effects model were used to combine direct and indirect evidence and by forming a connection network to compare various drug interventions simultaneously, and the multivariate meta-analysis was adopted. Conventional pairwise meta-analyses across comparisons available for each contrast were conducted; placebo (PLB) or standard neurosurgical treatment (SNT) group was the designated control group for pairwise meta-analysis. A network plot was drawn to briefly present all the available evidence of each treatment therapy, with distinct treatment expressed by different nodes, and trials are expressed by lines joining appropriate nodes. Then, a funnel plot was drawn, which was analyzed by Egger’s test (Seagroatt and Stratton, 1998) to detect any types of bias, such as small sample effect or selective reporting bias. The above analyses were performed in STATA, version 16.0 (College Station, TX, USA).

In order to estimate the unique and primary outcome, our NMA was performed non-informatively prior to distributions and by using the Markov chain Monte Carlo (MCMC) method under a Bayesian framework (Mavridis and Salanti, 2013; Green and Worden, 2015) in OpenBUGS (version 3.2.3 rev 1012). Odds ratio (OR) and 95% CrI were calculated as the pooled relative effect and estimate uncertainly, respectively. Under the circumstances of randomly selecting the state, three Markov chains were selected for the initial value setting, the number of iterations for the initial update was set as 50,000 for each chain, and the first 10,000 annealings were discarded to eliminate the influence of the initial value bias, and sampling started after 10,001. The iterative convergence was evaluated by Gelman–Rubin–Brooks diagnosis. Random- or fixed-effect models were selected regarding the deviance information criterion (DIC) value, and it is generally believed that the DIC value is as small as possible. The details of the Open BUGS code are presented in Supplementary Material Bayesian categorical code. The treatment rank probability was calculated, and the surface under the cumulative ranking curve (SUCRA) was generated to display the cumulative ranking probability plots of different interventions included. A higher SUCRA value indicates a better intervention effect. For the closed loop formed by the intervention in the entire network, the “node-splitting” technique (van Valkenhoef et al., 2016) was used to test the inconsistency, and *p*-value > 0.05 indicates no inconsistency (Stang, 2010).

## Results

### Baseline Characteristics

**FIGURE 1:**
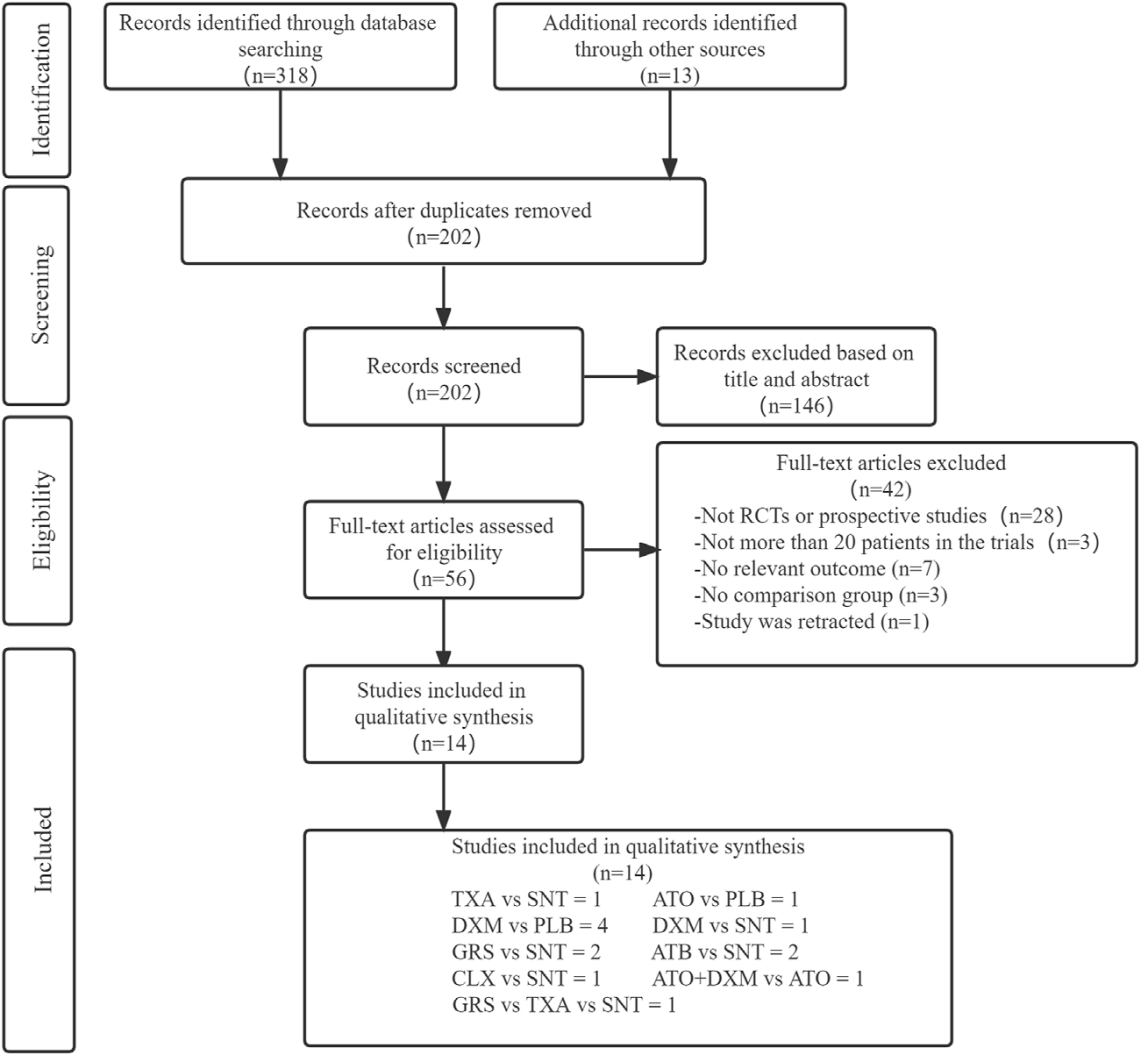
Screening chart graph. Abbreviations: TXA, tranexamic acid; DXM, dexamethasone; ATO, atorvastatin; GRS, goreisan; CLX, celecoxib; ATO+DXM, atorvastatin plus dexamethasone; ATB, antithrombotic; PLB or SNT, placebo or standard neurosurgical treatment; RCT, randomized controlled study.

Through database search, 318 articles were preliminarily screened, and additional 13 articles were obtained by tracking the references of the originally screened articles. Then 128 duplicates and other 146 articles were eliminated after reading the title and abstract. After full-text examination of the remaining articles, 42 articles were excluded, as 28 articles were not RCTs or prospective studies, 3 articles did not include more than 20 patients, 7 articles were without relevant main outcome or reported data that cannot be extracted, 3 articles were without a control group, and 1 article was retracted. Finally, 14 articles (Sun et al., 2005; Prud’homme et al., 2016; Schaumann et al., 2016; Brennan et al., 2017; Jiang et al., 2018; Katayama et al., 2018; Wan et al., 2020; Hutchinson et al., 2020; Wang et al., 2020; Mebberson et al., 2020; Yamada and Natori, 2020; Fujisawa et al., 2021; Tariq and Bhatti, 2021; Poon et al., 2021), including 6 drug interventions and involving a total of 3,456 patients, were included in our NMA. Figure 1 shows the processing of literature selection.

The included studies were published from 2005 to 2021. Table 1 summarizes the major characteristics of participants and drug interventions of the 14 included trials. The participants included in each study were all CSDH patients. Twelve articles were RCTs, and 2 articles were prospective studies. The duration of treatments varied from 2 to 12 weeks. According to available data, 71.88% of patients were male, and all included patients’ mean age ranged from 63.0 to 79.2 years. Three of the included articles used conservative basic treatment in patients with CSDH, burr hole alone was used in 5 articles as their basic treatment, burr hole or craniotomy was used in 4 articles, and the remaining 2 articles included both conservative and burr-hole treatment in patients.

**TABLE 1:** Characteristics of included studies.

Publication	Study design	Treatments and sample size	Mean age (years, ±SD)	Gender (male)	Basic treatment	Doses	Treatment duration	Recruiting area
Wan 2020	RCT	TXA = 41 versus SNT = 49	72.02 ± 11.79 versus 69.57 ± 13.69	60 (66.7)	Burr hole or craniotomy	500 mg twice daily	3 weeks	Singapore
Jiang 2018	RCT	ATO = 98 versus PLB = 98	63 ± 12.84 versus 67 ± 12.64	169 (86.2)	Conservative	20 mg nightly	8 weeks	China
Hutchinson 2020	RCT	DXM = 375 versus PLB = 373	74.5 ± 11.8 versus 74.3 ± 11	554 (74.1)	COB	Total 124 mg of 2 weeks※	2 weeks	United Kingdom
Prud’homme 2015	RCT	DXM = 10 versus PLB = 10	69.4 ± 8.8 versus 72.3 ± 6.3	18 (90.0)	Conservative	4 mg three times a day	3 weeks	Canada
Mebberson 2019	RCT	DXM = 23 versus PLB = 24	73.39 ± 15.4 versus 75.13 ± 15.5	34 (72.3)	Burr hole or craniotomy	Total 128 mg of 2 weeks^¶^	2 weeks	Australia
Sun 2005	Prospective	DXM = 95 versus PLB = 17	73.85 ± 10.6	63 (56.3)	COB	4 mg four times a day	3 weeks	Hong Kong
Katayama 2018	RCT	GRS = 92 versus SNT = 88	75.8 ± 9.53 versus 75.9 ± 8.08	137 (76.1)	Burr hole	750 mg three times per day	12 weeks	Japan
Yamada 2019	RCT	GRS = 78 versus TXA = 72 versus SNT = 82	79.2 ± 8.7 versus 78.2 ± 9.2 versus 78.8 ± 10.8	150 (64.7)	Burr hole	750 mg three times per day	12 weeks	Japan
Schaumann 2016	RCT	CLX = 10 versus SNT = 13	68.0 versus 71.0	16 (69.6)	Burr hole	200 mg twice daily	4 weeks	Germany
Fujisawa 2020	RCT	GRS = 104 versus SNT = 104	74 ± 3.38 versus 74 ± 2.99	153 (73.6)	Burr hole	750 mg three times per day	12 weeks	Japan
Wang 2019	RCT	ATO+DXM = 104 versus ATO = 104	69.37 ± 10.9 versus 63.83 ± 13.73	45 (75.0)	Conservative	Special dose*	5 weeks	China
Brennan 2016	Prospective	ATB = 161 versus SNT = 523	76.49 ± 12.7	465 (68)	Burr hole or craniotomy	NP	1–44 d	United Kingdom
Tariq 2021	RCT	DXM = 46 versus SNT = 46	62.7 ± 12.9 versus 63.8 ± 12.7	67 (72.8)	Burr hole	Total 134 mg of 2 weeks#	2 weeks	Pakistan
Poon 2018	Prospective	ATB = 328 versus SNT = 436	78.9 ± 1.8 versus 74.9 ± 3.5	553 (72.4)	Burr hole or craniotomy	NP	NP	Sweden

Note. TXA, tranexamic acid; DXM, dexamethasone; ATO, atorvastatin; GRS, goreisan; CLX, celecoxib; ATO+DXM, atorvastatin plus dexamethasone; ATB, antithrombotic; PLB or SNT, placebo or standard neurosurgical treatment; RCT, randomized controlled study; COB, conservative or burr hole; NP, not reported.

^※^Total 124 mg of 2 weeks: oral 8 mg twice daily on days 1–3, then 6 mg twice daily on days 4–6, then 4 mg twice daily on days 7–9, then 2 mg twice daily on days 10–12, and then 2 mg once daily on days 13 and 14.

^**¶**^Total 128 mg of 2 weeks: oral 4 mg as 1 capsule 4 times a day for 3 days, then 1 capsule 3 times a day for 3 days, then 1 capsule twice daily for 3 days, and finally 1 capsule daily thereafter.

^*^Special dose: DXM 2.25 mg daily for 2 weeks followed by 0.75 mg twice daily for 2 weeks and subsequently at 0.75 mg once a day for 1 week and ATO 20 mg nightly.

^**#**^Total 134 mg of 2 weeks: 16 mg dexamethasone was administered in 4 divided doses per day for the first 2 postoperative days and tapered in 3-mg decrements every 3 days.

We extracted the relevant data of the included trials and summarized it in Table 2. The results showed that all trials reported the recurrence rates, and the overall recurrence rate was about 6% (range from 0% to 11.2%) in the intervention group and 13.6% (range from 4.3% to 30%) in the control group.

**TABLE 2:** Recurrence rates of included studies in our NMA.

Publication	Recurrence rates (%)		OR or HR (95%CI)	*p*-Value
	IG	CG		
Wan 2020	TXA: 4.8	SNT: 10.2	0.51 (0.11–2.47)	0.221
Jiang 2018^#^	ATO: 11.2	PLB: 23.5	0.47 (0.24–2.92)	0.03
Hutchinson 2020	DXM: 1.7	PLB: 7.1	NP	NP
Prud’homme 2015	DXM: 10.0	PLB: 30.0	NP	NP
Mebberson 2019	DXM: 0	PLB: 20.8	NP	0.049
Sun 2005	DXM: 4.2	PLB: 23.5	NP	NP
Katayama 2018	GRS: 9.8	SNT: 12.5	NP	0.56
Yamada 2019^*^	TXA: 1.4	SNT: 9.8	NP	0.083
	GRS: 9.0			
Schaumann 2016	CLX: 10.0	SNT: 10.0	NP	NP
Fujisawa 2020	GRS: 5.8	SNT: 5.8	0.42 (0.15–1.17)	0.09
Wang 2019	ATO+DXM: 3.3	ATO: 13.3	NP	0.353
Brennan 2016	ATB: 6.8	SNT: 8.9	NP	NP
Tariq 2021	DXM: 2.2	SNT: 4.3	NP	0.557
Poon 2018	ATB: 9.9	SNT: 10.1	NP	0.93

Note. TXA, tranexamic acid; DXM, dexamethasone; ATO, atorvastatin; GRS, goreisan; CLX, celecoxib; ATO+DXM, atorvastatin plus dexamethasone; ATB, antithrombotic; PLB or SNT, placebo or standard neurosurgical treatment; IG, intervention group; CG, control group; OR, odds ratio; HR, hazard ratio; NP, not reported.

^*^Yamada 2019: this is a three-arm clinical trial.

^#^Jiang 2018: this trial used hazard ratio.

### Risk of Bias Quality Assessment

**FIGURE 2:**
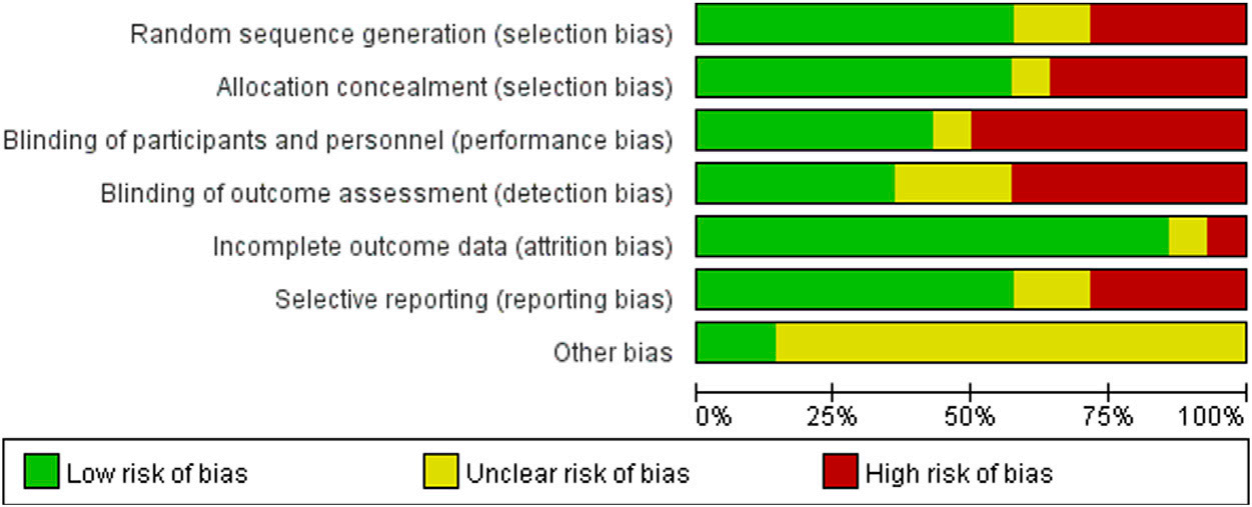
Risk of bias assessment.

**FIGURE 3:**
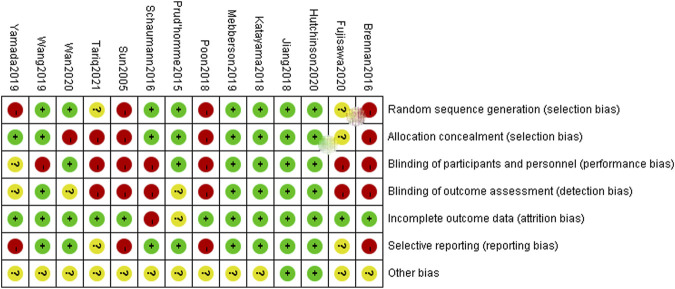
Summary of risk of bias assessment. Risk of bias of included randomized controlled trials or prospective trials (review authors’ judgments about each risk of bias item for each included study: +, low risk; −, high risk; ?, unclear risk).

Within the 14 included trials, 7 trials described in detail the random sequence generation and their approach of allocation concealment, and 6 trials described the blinding methods of participants and personnel. Four studies may have selective reporting bias, and only 2 studies may have incomplete data. The individual bias and overall bias of study-level quality are summarized in Figures 2, 3, respectively.

### Pairwise Meta-Analysis and Network Meta-Analysis Results

**FIGURE 4:**
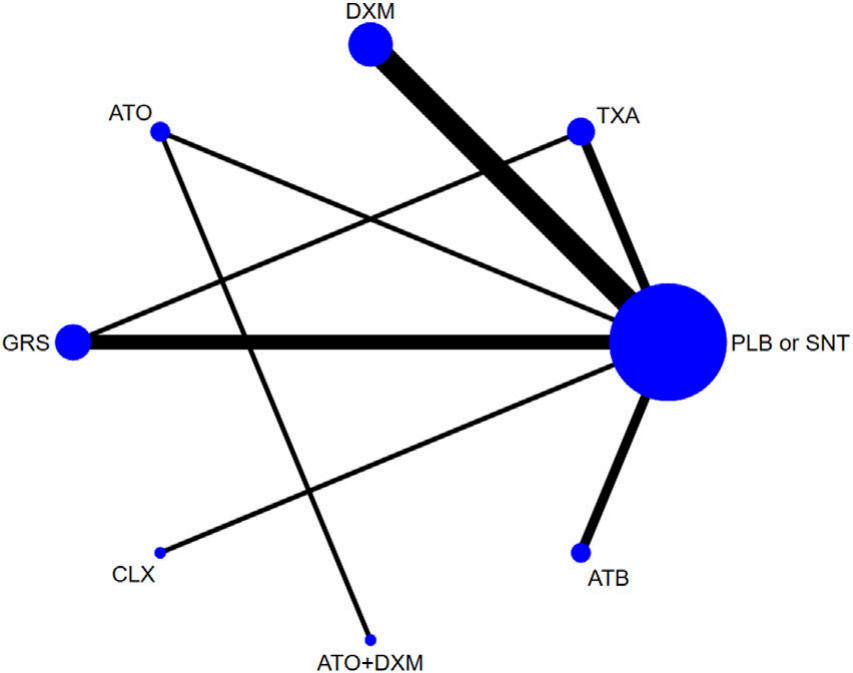
Network plot. Abbreviations: TXA, tranexamic acid; DXM, dexamethasone; ATO, atorvastatin; GRS, goreisan; CLX, celecoxib; ATO+DXM, atorvastatin plus dexamethasone; ATB, antithrombotic; PLB or SNT, placebo or standard neurosurgical treatment.

As shown in Figure 4, our network plot illustrates those comparisons between the seven drug intervention groups. Table 3 summarizes that dexamethasone (DXM) was most frequently included with 5 arms (*n* = 549), followed by GRS involving 3 arms (*n* = 274), atorvastatin (ATO) involving 1 arm (*n* = 98), TXA involving 2 arms (*n* = 113), antithrombotic (ATB) involving 2 arms (*n* = 489), celecoxib (CLX) involving 1 arm (*n* = 10), and ATO+DXM involving 1 arm (*n* = 104), among which 2 studies were direct trials and 1 of them was a three-arm clinical trial.

**TABLE 3:** Efficacy of different intervention drugs compared to designated control group.

Intervention drugs	Number of arms	Number of patients (IG vs. DCG^*^)		OR (95%CrI)	SUCRA (%)
ATO+DXM	1	104 vs. 98	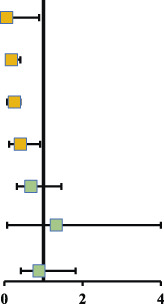	0.06 (0.01, 0.89)	0.89 (0.29, 1.00)
DXM	5	549 vs. 470		0.18 (0.07, 0.41)	0.78 (0.57, 1.00)
TXA	2	113 vs. 121		0.26 (0.07, 0.41)	0.75 (0.43, 1.00)
ATO	1	98 vs. 98		0.41 (0.12, 0.90)	0.54 (0.14, 0.86)
GRS	3	274 vs. 274		0.68 (0.32, 1.46)	0.36 (0.00, 0.71)
CLX	1	10 vs. 13		1.33 (0.77, 24.3)	0.25 (0.00, 1.00)
ATB	2	489 vs. 959		0.89 (0.42, 1.82)	0.24 (0.00, 0.57)

Note. TXA, tranexamic acid; DXM, dexamethasone; ATO, atorvastatin; GRS, goreisan; CLX, celecoxib; ATO+DXM, atorvastatin plus dexamethasone; ATB, antithrombotic; PLB or SNT, placebo or standard neurosurgical treatment; IG, intervention group; DCG, designated control group; CrI, credibility interval; SUCRA, the surface under the cumulative ranking curve; OR, odds ratio.

^*^DCG: PLB or SNT was the designated control group for pairwise meta-analysis.

**FIGURE 5:**
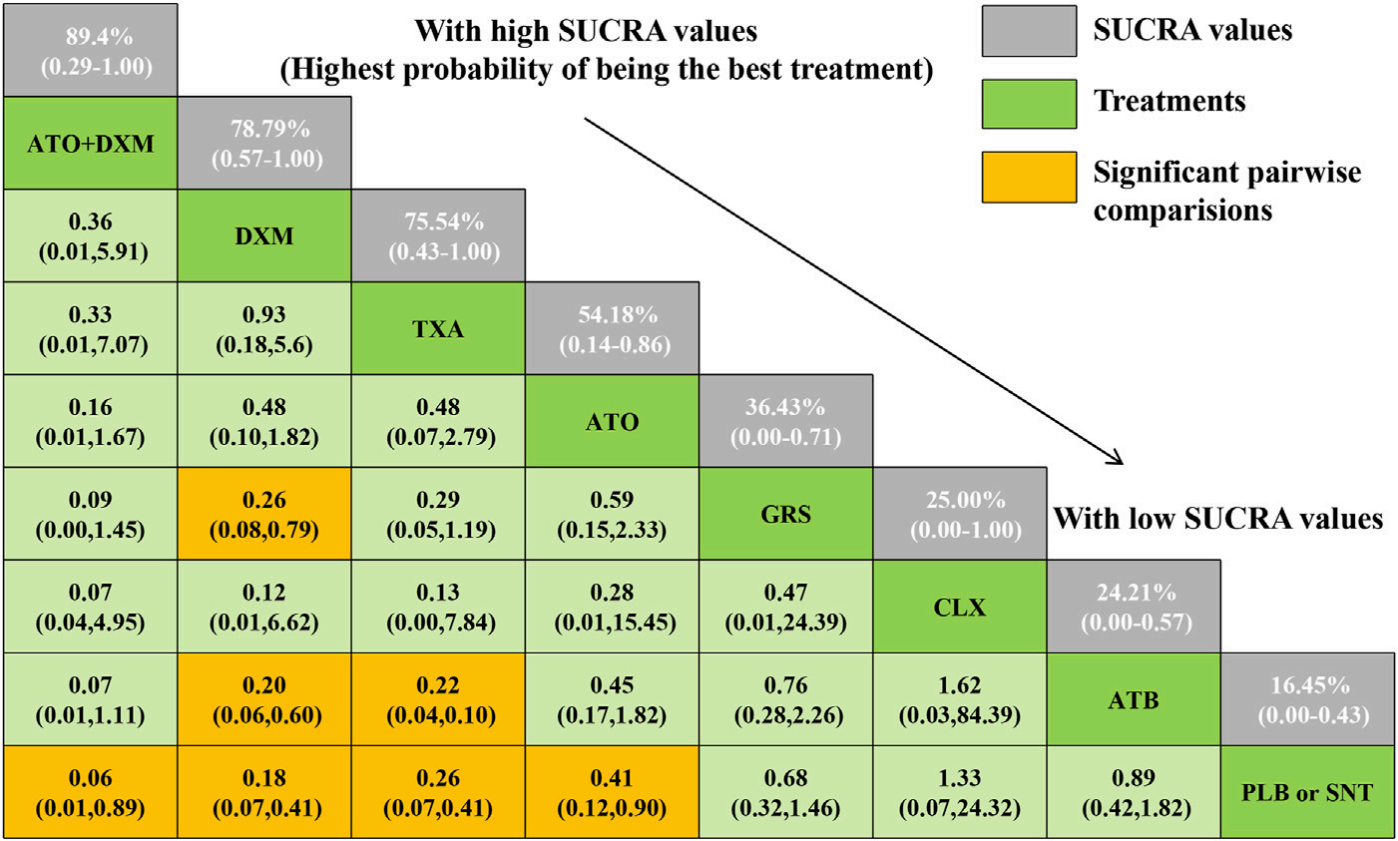
Relative effect sizes of efficacy at posttreatment according to network meta-analysis. Abbreviation: SUCRA, the surface under the cumulative ranking curve.

As illustrated in Figure 5, a total of 4 drugs were statistically significantly superior to the PLB or SNT group, including ATO+DXM (OR = 0.06, 95% CrI 0.01, 0.89), DXM (OR = 0.18, 95% CrI 0.07, 0.41), TXA (OR = 0.26, 95% CrI 0.07, 0.41), and ATO (OR = 0.41, 95% CrI 0.12, 0.90). In addition, the efficacy of DXM and TXA was significantly higher than that of ATB, and the efficacy of DXM was also significantly higher than that of GRS. The remaining drug interventions (GRS: OR = 0.68, 95% CrI 0.32, 1.46; CLX: OR = 1.33, 95% CrI 0.07, 24.32; ATB: OR = 0.89, 95% CrI 0.42, 1.82) were more likely to improve the recurrence in the patients compared with the PLB or SNT group, but these differences were not statistically significant.

**FIGURE 6:**
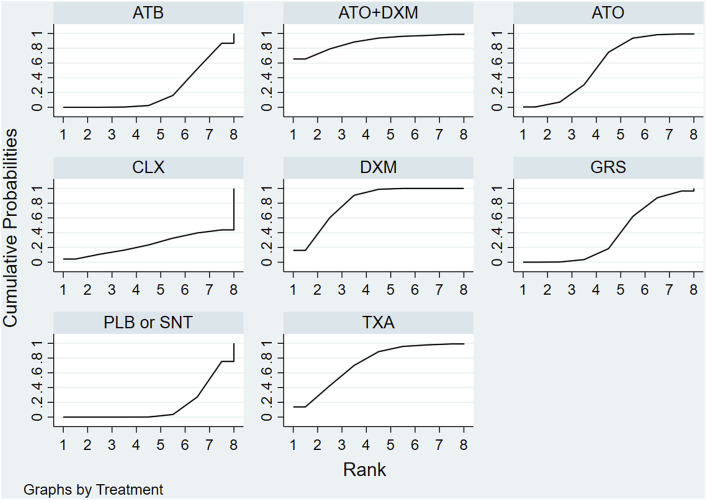
SUCRA plot. The surface under the cumulative ranking curve (SUCRA) was generated to display a simple numerical statistical cumulative ranking probability plot of various interventions. SUCRA is 1 if treatment is certainly at the highest level or highly effective, while zero if it undoubtedly means that the treatment has the worst effect. Abbreviations: TXA, tranexamic acid; DXM, dexamethasone; ATO, atorvastatin; GRS, goreisan; CLX, celecoxib; ATO+DXM, atorvastatin plus dexamethasone; ATB, antithrombotic; PLB or SNT, placebo or standard neurosurgical treatment; SUCRA, the surface under the cumulative ranking curve.

The SUCRA line was plotted to rank each drug intervention (shown in Figures 5 and 6), which showed that ATO+DXM had the highest probability of improving the recurrence in CSDH patients (SUCRA = 89.40%, 95% CrI 0.29, 1.00), while DXM (SUCRA = 78.79%, 95% CrI 0.57, 1.00), TXA (SUCRA = 75.74%, 95% CrI 0.43, 1.00), and ATO (SUCRA = 54.18%, 95% CrI 0.14, 0.86) also had a good ranking among the 8 interventions compared with other 7 drug interventions. The remaining GRS (SUCRA = 36.43%, 95% CrI 0.00, 0.71), CLX (SUCRA = 25.00%, 95% CrI 0.00, 1.00), ATB (SUCRA = 24.21%, 95% CrI 0.00, 0.57), and PLB or SNT (SUCRA = 16.45%, 95% CrI 0.00, 0.43) had an inferior ranking. Testing for inconsistency resulted in no statistical significance (*p*-value = 0.4672), and there also was no statistically significant inconsistency between direct and indirect comparisons tested by node-splitting technique (PLB or SNT vs. TXA *p*-value = 0.399, PLB or SNT vs. ATO *p*-value = 0.990, PLB or SNT vs. GRS *p*-value = 0.307, TXA vs. GRS *p*-value = 0.258, ATO vs. ATO+DXM *p*-value = 0.990).

**FIGURE 7:**
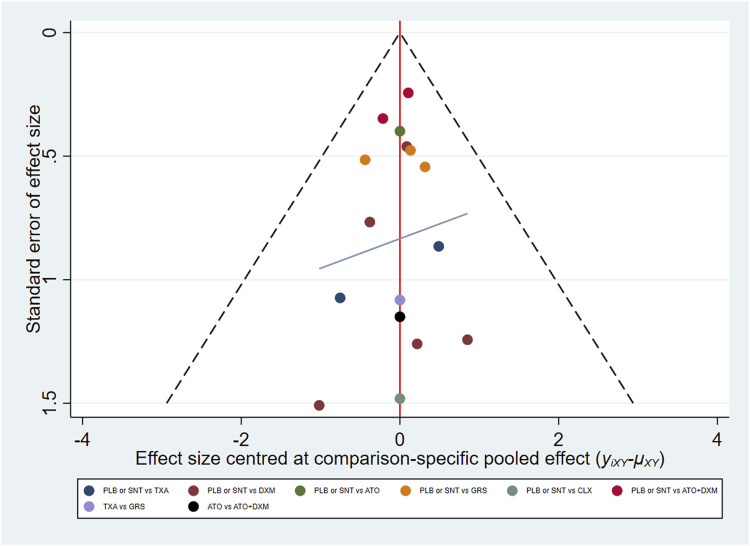
Funnel plot. Red line suggests the null hypothesis that study-specific effect sizes do not differ from respective comparison-specific pooled effect estimates. Different colors represent different comparisons. Abbreviations: TXA, tranexamic acid; DXM, dexamethasone; ATO, atorvastatin; GRS, goreisan; CLX, celecoxib; ATO+DXM, atorvastatin plus dexamethasone; ATB, antithrombotic; PLB or SNT, placebo or standard neurosurgical treatment.

The funnel plot shows that the distribution of some asymmetric scattering points in this inverted funnel plot indicates that some publication bias may be generated (Figure 7).

## Discussion

The results of our NMA summarized the available data, suggesting that the optimal drug intervention for CSDH to reduce recurrence is ATO+DXM (OR = 0.06, 95% CrI 0.01, 0.89, SUCRA = 89.40%, 95% CrI 0.29, 1.00), and we also observed corresponding definite curative effectiveness of DXM, TXA, and ATO on the recurrence improvement of CSDH. The remaining three drug therapies also showed better efficacy in improving recurrence compared with the PLB or SNT group (the efficacy was ranked GRS, CLX, and ATB from high to low), but this difference was not significant, and the results need to be discussed cautiously. These results may provide useful evidence for clinicians to prescribe effective drugs for patients with CSDH.

Presently, craniotomy or burr-hole craniotomy to remove hematoma was still the main treatment for symptomatic patients with CSDH. Nevertheless, these surgeries are also accompanied by a high rate of recurrence (10%) (Santarius et al., 2009; Soleman et al., 2019); besides, even conservative patients with less bleeding and who are asymptomatic still face the possibility of subdural progressive recurrent bleeding. Under these circumstances, regardless of whether surgery was performed, patients with CSDH also should undergo drug treatment that can effectively prevent recurrence.

An excessive inflammatory reaction reported to assist in outer membrane formation of CSDH was considered a potential mechanism to explain the recurrence (Edlmann et al., 2017). Therefore, inhibition of excessive inflammatory response and promotion of neovascularization have become therapeutic strategies to promote CSDH absorption. In a preliminary study, ATO, as an inhibitor of 3-hydroxy-3-methylglutaryl-coenzyme A reductase, was first reported to lead to a hematoma volume reduction in CSDH patients (Wang et al., 2014). Then, ATO was demonstrated to reduce inflammation and hematoma in an SDH rat model (Li et al., 2014). It is shown that statins have an anti-inflammatory effect (reducing MCP1 and TNF-α) and can mobilize endothelial progenitor cells for vascular repair in previous literature (Araújo et al., 2010; Lin et al., 2014). ATO did have definite curative effectiveness to reduce the incidence of recurrence, which requires surgery in our NMA. Our conclusion is consistent with that of a recent meta-analysis of 6 studies comprising 756 patients with CSDH, which also suggested that ATO could improve prognosis and neurological recovery (He et al., 2021). Additionally, some studies even suggest that ATO is as effective as surgery in patients with mild CSDH (Shofty and Grossman, 2016; Soleman et al., 2017). Currently, low-dose ATO has been used by many neurosurgeons to promote CSDH absorption and prevent the recurrence of CSDH (Wang et al., 2014; Chan et al., 2017; Tang et al., 2018). It is recommended that future clinical trials of ATO focus more on its improvement in life quality and outcomes in patients with CSDH.

DXM was shown to better improve recurrence as compared to ATO in our NMA, maybe because it can provide a stronger anti-inflammatory effect and antiangiogenic effects (Sun et al., 2005). It is known that DXM, as a synthetic version of naturally occurring corticosteroid hormone, inhibits the aggregation, phagocytosis, and release of inflammatory mediators by immune-inflammatory cells. Glover et al., in their early studies, indicated that DXM was demonstrated to cause significantly lighter and smaller blood clots in CSDH, and the underlying reason may be the inhibition of inflammatory response, which leads to improper development of the outer membrane (Glover and Labadie, 1976). Two recent meta-analyses (Holl et al., 2019; Shrestha et al., 2021) also indicated that treatment with DXM was associated with a lesser recurrence of CSDH, but the effect of DXM to improve neurological outcomes and reduce mortality was not observed. Moreover, it should be noted that most of their included studies were observational and nonrandomized; thus, the credibility of their conclusions is relatively insufficient. However, one of the major defects of DXM therapy is the significant side effect of intravenous application, which may outweigh the benefits, as retrospective trials indicated that the use of high-dose DXM (6–8 mg/day) could only save 17% of patients with CSDH from the operation but significantly increase treatment complications (Miah et al., 2020). Therefore, low-dose DXM treatment is always used clinically, and the duration should be no more than 4 weeks, but the application of DXM is still inevitably accompanied by side effects. The research of Wang et al. (2021)indicated that DXM increased the risk of all-cause mortality (relative risk (RR) = 1.96, 95% CrI 1.20, 3.28) of CSDH, and the adverse events resulting from DXM treatment were generally serious even with low doses.

Additionally, our studies found that the low-dose DXM combined with ATO resulted in the best effectiveness concerning reducing reoperation as compared to any other drug interventions. The intuitive reason was that low doses and short-term use of DXM in combination with ATO can further enhance the inhibition of inflammatory reaction, thereby reducing vascular leakage and decreasing the risk of hematoma expansion. The recent study by Gong et al. (2021) for the first time provided evidence that the underlying mechanisms of the improved efficacy of this combined therapy were primarily by increasing the presence of ATO in hematoma and macrophages and by regulating the alteration of the macrophage phenotype, promoting the transition from the proinflammatory phenotype to the anti-inflammatory phenotype. As a result, this combination therapy showed a better effect to correct imbalances between the CSDH injury factor and repair factor. Besides, this combination also can simultaneously avoid the adverse effects of large doses and long-term use of DXM. Interestingly, CLX (a selective COX-2 inhibition) works by the same mechanism, which is to inhibit inflammation, but fails to improve CSDH recurrence. Whether other anti-inflammatory drugs in general (except for ATO and DXM) can prevent the development of CSDH significantly raises an important question, but more trials in this field are needed. Equally interesting is the observation that the statistical results of ATB (OR = 0.89, 95% CrI 0.42, 1.82) were more likely to improve the recurrence in CSDH patients than the PLB or SNT group; this difference was not statistically significant, and the results obviously need to be discussed cautiously. Given the ATB sample size, the included studies of ATB were only prospective trials, and the difference was not statistically significant; therefore, the results interpreted here indicate more that ATB may not increase the risk of recurrence, instead of reducing recurrence significantly. In addition, whether ATB increases the risk of recurrence of CSDH remains controversial in the current literature. The application of ATB (optimal dose, withdrawal time, etc.) still needs to be evaluated in more well-designed clinical trials in the future.

Antifibrinolytic therapy of CSDH has attracted growing attention. Some studies have shown that in patients with CSDH hematoma fluid, outer membrane organization fibrinolytic enzyme activation, and fibrin degradation products, thrombosis regulatory protein increased significantly and repeatedly prompted high fibrinolytic associated with blood vessel leak blood (Fujisawa et al., 1995; Murakami et al., 2002; Katano et al., 2006; Shim et al., 2007). Antifibrinolytic drugs by inhibition of the fibrinolytic enzyme activation and fibrinolytic enzyme activity stop the bleeding. Currently, traditional meta-analysis to assess the efficacy of TXA in reducing recurrence of CSDH has not yet appeared. Some retrospective studies have concluded that TXA, as an antifibrinolytic drug, can be used to promote CSDH hematoma absorption and reduce recurrence (Kageyama et al., 2013; Stary et al., 2016; Tanweer et al., 2016). TXA also can play an indirect anti-inflammatory role through the kallikrein–kinin pathway (Hunt, 2015). Our NMA results showed that TXA is beneficial for the reduction of recurrence (SUCRA = 75.74%, 95% CrI 0.43, 1.00), which is consistent with the above studies. In addition, in our research, the effectiveness of TXA is weaker than that of DXM but higher than that of ATO.

GRS is an herbal medicine prescription developed as a new alternative treatment in patients with CSDH. It works by inhibiting aquaporin-4, which is expressed in the outer membrane of the CSDH (Kwon et al., 2019). GRS prevents the inflow of fluid into the hematoma, thereby preventing the development and recurrence of hematoma. Our NMA showed that GRS did not have a definite efficacy improvement in recurrence, and currently, relevant meta-analysis is also lacking in comparison with our results.

The drug interventions applied to CSDH are complex and multifaceted. The number of existing comprehensive and rigorously compared treatment studies for this disease is still largely insufficient. Recently, Wang et al. (2021) analyzed the efficacy and safety of five drug treatments on the drug management of CSDH and concluded that DXM was the best treatment to reduce recurrence. Our NMA included more drug interventions (ATO+DXM, CLX, and ATB) in conducting further analysis for the recurrence improvement, which showed that DXM did have a definite effect of reducing recurrence, but ATO in combination with DXM showed a stronger effect in this particular aspect; these are the strength of our research. But remarkably, this combination therapy may be accompanied by more adverse complications, and the analysis of Wang et al. of the safety of drugs in CSDH is clearly useful and necessary. Regrettably, the optimal dosage and duration that could provide the best benefit without serious adverse effects of DXM in CSDH remain unclear. For the application of DXM, we need to be extremely cautious. In addition, due to the limited evidence-based data, ATO+DXM therapy still needs to be further evaluated by more RCTs. Similarly, under the situation of considering the safety in advance, rigorous RCTs also need to be designed in the future to evaluate other drug combinations (e.g., DXM+GRS) that may have potential benefits.

The greatest advantage of our NMA is that we combined and summarized all eligible studies to make a comprehensive effectiveness comparison of drug interventions in patients with CSDH, thus making up for the lack of contrast studies of many drugs that are sufficiently innovative and have great clinical significance. However, the limitations of our NMA also need to be acknowledged. First, there are not enough RCTs or prospective studies on CLX, ATO+DXM, and other drug interventions, so the evidence based on its efficacy is limited, which may make it difficult for our NMA to draw a conclusion. Second, we did not analyze the side effects and outcomes of interventions, which also could influence clinical treatment options. Finally, the low quality of several trials may potentially threaten the validity of our NMA.

## Conclusion

To sum up, our NMA concluded that ATO+DXM, DXM, ATO, and TXA had definite curative efficacy in improving the recurrence in CSDH patients. Among them, ATO+DXM is the optimal drug intervention in this particular population to reduce recurrence. At the same time, the evidence from our NMA also can guide the development of clinical guidelines and thus help clinicians make more effective and appropriate decisions in clinical practice. Moreover, multicenter RCTs are still needed to evaluate the role of various drug interventions in improving neurological function or outcome.

## Data Availability

The original contributions presented in the study are included in the article/Supplementary Material, further inquiries can be directed to the corresponding authors.

## References

[B1] Araújo F. A. Rocha M. A. Mendes J. B. Andrade S. P. (2010). Atorvastatin Inhibits Inflammatory Angiogenesis in Mice through Down Regulation of VEGF, TNF-Alpha and TGF-Beta1. Biomed. Pharmacother. 64 (1), 29–34. 10.1016/j.biopha.2009.03.003 19811885

[B2] Balser D. Farooq S. Mehmood T. Reyes M. Samadani U. (2015). Actual and Projected Incidence Rates for Chronic Subdural Hematomas in United States Veterans Administration and Civilian Populations. J. Neurosurg. 123 (5), 1209–1215. 10.3171/2014.9.JNS141550 25794342 PMC4575892

[B3] Brennan P. M. Kolias A. G. Joannides A. J. Shapey J. Marcus H. J. Gregson B. A. (2017). The Management and Outcome for Patients with Chronic Subdural Hematoma: a Prospective, Multicenter, Observational Cohort Study in the United Kingdom. J. Neurosurg. 127 (4), 1–8. 10.3171/2016.8.JNS16134.test 28306417

[B4] Chan D. Y. Chan D. T. Sun T. F. Ng S. C. Wong G. K. Poon W. S. (2017). The Use of Atorvastatin for Chronic Subdural Haematoma: a Retrospective Cohort Comparison Study(). Br. J. Neurosurg. 31 (1), 72–77. 10.1080/02688697.2016.1208806 27881024

[B5] Cochrane Handbook for Systematic Reviews of Interventions. Chichester, United Kingdom: John Wiley & Sons. 2011.

[B6] de Faria J. L. da Silva Brito J. Costa E Silva L. T. Kilesse C. T. S. M. de Souza N. B. Pereira C. U. (2021). Tranexamic Acid in Neurosurgery: a Controversy Indication-Review. Neurosurg. Rev. 44 (3), 1287–1298. 10.1007/s10143-020-01324-0 32556832

[B7] Edlmann E. Giorgi-Coll S. Whitfield P. C. Carpenter K. L. H. Hutchinson P. J. (2017). Pathophysiology of Chronic Subdural Haematoma: Inflammation, Angiogenesis and Implications for Pharmacotherapy. J. Neuroinflammation 14 (1), 108. 10.1186/s12974-017-0881-y 28558815 PMC5450087

[B8] Frati A. Salvati M. Mainiero F. Ippoliti F. Rocchi G. Raco A. (2004). Inflammation Markers and Risk Factors for Recurrence in 35 Patients with a Posttraumatic Chronic Subdural Hematoma: a Prospective Study. J. Neurosurg. 100 (1), 24–32. 10.3171/jns.2004.100.1.0024 14743908

[B9] Fujisawa H. Ito H. Kashiwagi S. Nomura S. Toyosawa M. (1995). Kallikrein-kinin System in Chronic Subdural Haematomas: its Roles in Vascular Permeability and Regulation of Fibrinolysis and Coagulation. J. Neurol. Neurosurg. Psychiatry 59 (4), 388–394. 10.1136/jnnp.59.4.388 7561918 PMC486075

[B10] Fujisawa N. Oya S. Yoshida S. Tsuchiya T. Nakamura T. Indo M. (2021). A Prospective Randomized Study on the Preventive Effect of Japanese Herbal Kampo Medicine Goreisan for Recurrence of Chronic Subdural Hematoma. Neurol. Med. Chir (Tokyo) 61 (1), 12–20. 10.2176/nmc.oa.2020-0287 33208583 PMC7812313

[B11] Glover D. Labadie E. L. (1976). Physiopathogenesis of Subdural Hematomas. Part 2: Inhibition of Growth of Experimental Hematomas with Dexamethasone. J. Neurosurg. 45 (4), 393–397. 10.3171/jns.1976.45.4.0393 956875

[B12] Gong Z. Zhan D. Nie M. Li X. Gao C. Liu X. (2021). Dexamethasone Enhances the Efficacy of Atorvastatin in Inhibiting Excessively Inflammation-Induced Abnormal Angiogenesis by Regulating Macrophages. J. Neuroinflammation 18 (1), 203. 10.1186/s12974-021-02257-1 34526068 PMC8444603

[B13] Green P. L. Worden K. (2015). Bayesian and Markov Chain Monte Carlo Methods for Identifying Nonlinear Systems in the Presence of Uncertainty. Philos. Trans. A. Math. Phys. Eng. Sci. 373. 10.1098/rsta.2014.0405 PMC4549940 26303916

[B14] He C. Xia P. Xu J. Chen L. Zhang Q. (2021). Evaluation of the Efficacy of Atorvastatin in the Treatment for Chronic Subdural Hematoma: a Meta-Analysis. Neurosurg. Rev. 44 (1), 479–484. 10.1007/s10143-019-01218-w 31953781

[B15] Hohenstein A. Erber R. Schilling L. Weigel R. (2005). Increased mRNA Expression of VEGF within the Hematoma and Imbalance of Angiopoietin-1 and -2 mRNA within the Neomembranes of Chronic Subdural Hematoma. J. Neurotrauma 22 (5), 518–528. 10.1089/neu.2005.22.518 15892598

[B16] Holl D. C. Volovici V. Dirven C. M. F. Peul W. C. van Kooten F. Jellema K. (2018). Pathophysiology and Nonsurgical Treatment of Chronic Subdural Hematoma: From Past to Present to Future. World Neurosurg. 116, 402–e2. 10.1016/j.wneu.2018.05.037 29772364

[B17] Holl D. C. Volovici V. Dirven C. M. F. van Kooten F. Miah I. P. Jellema K. (2019). Corticosteroid Treatment Compared with Surgery in Chronic Subdural Hematoma: a Systematic Review and Meta-Analysis. Acta Neurochir (Wien) 161 (6), 1231–1242. 10.1007/s00701-019-03881-w 30972566

[B18] Hunt B. J. (2015). The Current Place of Tranexamic Acid in the Management of Bleeding. Anaesthesia 70 Suppl 1 (Suppl. 1), 50e18–e18. 10.1111/anae.12910 25440395

[B19] Hutchinson P. J. Edlmann E. Bulters D. Zolnourian A. Holton P. Suttner N. (2020). Trial of Dexamethasone for Chronic Subdural Hematoma. N. Engl. J. Med. 383 (27), 2616–2627. 10.1056/NEJMoa2020473 33326713

[B20] Hutton B. Salanti G. Caldwell D. M. Chaimani A. Schmid C. H. Cameron C. (2015). The PRISMA Extension Statement for Reporting of Systematic Reviews Incorporating Network Meta-Analyses of Health Care Interventions: Checklist and Explanations. Ann. Intern. Med. 162 (11), 777–784. 10.7326/M14-2385 26030634

[B21] Jiang R. Zhao S. Wang R. Feng H. Zhang J. Li X. (2018). Safety and Efficacy of Atorvastatin for Chronic Subdural Hematoma in Chinese Patients: A Randomized ClinicalTrial. JAMA Neurol. 75 (11), 1338–1346. 10.1001/jamaneurol.2018.2030 30073290 PMC6248109

[B22] Kageyama H. Toyooka T. Tsuzuki N. Oka K. (2013). Nonsurgical Treatment of Chronic Subdural Hematoma with Tranexamic Acid. J. Neurosurg. 119 (2), 332–337. 10.3171/2013.3.JNS122162 23641825

[B23] Kalamatianos T. Stavrinou L. C. Koutsarnakis C. Psachoulia C. Sakas D. E. Stranjalis G. (2013). PlGF and sVEGFR-1 in Chronic Subdural Hematoma: Implications for Hematoma Development. J. Neurosurg. 118 (2), 353–357. 10.3171/2012.10.JNS12327 23140147

[B24] Katano H. Kamiya K. Mase M. Tanikawa M. Yamada K. (2006). Tissue Plasminogen Activator in Chronic Subdural Hematomas as a Predictor of Recurrence. J. Neurosurg. 104 (1), 79–84. 10.3171/jns.2006.104.1.79 16509150

[B25] Katayama K. Matsuda N. Kakuta K. Naraoka M. Takemura A. Hasegawa S. (2018). The Effect of Goreisan on the Prevention of Chronic Subdural Hematoma Recurrence: Multi-Center Randomized Controlled Study. J. Neurotrauma 35 (13), 1537–1542. 10.1089/neu.2017.5407 29444611

[B26] Kwon S. Jin C. Cho K. H. (2019). Oreongsan, an Herbal Medicine Prescription Developed as a New Alternative Treatment in Patients with Chronic Subdural Hematoma: a Narrative Review. Integr. Med. Res. 8 (1), 26–30. 10.1016/j.imr.2018.11.003 30705821 PMC6348234

[B27] Li T. Wang D. Tian Y. Yu H. Wang Y. Quan W. (2014). Effects of Atorvastatin on the Inflammation Regulation and Elimination of Subdural Hematoma in Rats. J. Neurol. Sci. 341 (1-2), 88–96. 10.1016/j.jns.2014.04.009 24774750

[B28] Lin L. Y. Huang C. C. Chen J. S. Wu T. C. Leu H. B. Huang P. H. (2014). Effects of Pitavastatin versus Atorvastatin on the Peripheral Endothelial Progenitor Cells and Vascular Endothelial Growth Factor in High-Risk Patients: a Pilot Prospective, Double-Blind, Randomized Study. Cardiovasc. Diabetol. 13, 111. 10.1186/s12933-014-0111-1 25027585 PMC4223413

[B29] Mavridis D. Salanti G. (2013). A Practical Introduction to Multivariate Meta-Analysis. Stat. Methods Med. Res. 22 (2), 133–158. 10.1177/0962280211432219 22275379

[B30] Mebberson K. Colditz M. Marshman L. A. G. Thomas P. A. W. Mitchell P. S. Robertson K. (2020). Prospective Randomized Placebo-Controlled Double-Blind Clinical Study of Adjuvant Dexamethasone with Surgery for Chronic Subdural Haematoma with post-operative Subdural Drainage: Interim Analysis. J. Clin. Neurosci. 71, 153–157. 10.1016/j.jocn.2019.08.095 31492485

[B31] Miah I. P. Herklots M. Roks G. Peul W. C. Walchenbach R. Dammers R. (2020). Dexamethasone Therapy in Symptomatic Chronic Subdural Hematoma (DECSA-R): A Retrospective Evaluation of Initial Corticosteroid Therapy versus Primary Surgery. J. Neurotrauma 37 (2), 366–372. 10.1089/neu.2019.6541 31452450

[B32] Murakami H. Hirose Y. Sagoh M. Shimizu K. Kojima M. Gotoh K. (2002). Why Do Chronic Subdural Hematomas Continue to Grow Slowly and Not Coagulate? Role of Thrombomodulin in the Mechanism. J. Neurosurg. 96 (5), 877–884. 10.3171/jns.2002.96.5.0877 12005395

[B33] Poon M. T. C. Rea C. Kolias A. G. Brennan P. M. (2021). Influence of Antiplatelet and Anticoagulant Drug Use on Outcomes after Chronic Subdural Hematoma Drainage. J. Neurotrauma 38 (8), 1177–1184. 10.1089/neu.2018.6080 30526281 PMC8060161

[B34] Prud'homme M. Mathieu F. Marcotte N. Cottin S. (2016). A Pilot Placebo Controlled Randomized Trial of Dexamethasone for Chronic Subdural Hematoma. Can. J. Neurol. Sci. 43 (2), 284–290. 10.1017/cjn.2015.393 26853325

[B35] Rauhala M. Luoto T. M. Huhtala H. Iverson G. L. Niskakangas T. Öhman J. (2019). The Incidence of Chronic Subdural Hematomas from 1990 to 2015 in a Defined Finnish Population. J. Neurosurg. 132 (4), 1–11. 10.3171/2018.12.JNS183035 30901751

[B36] Santarius T. Kirkpatrick P. J. Ganesan D. Chia H. L. Jalloh I. Smielewski P. (2009). Use of Drains versus No Drains after Burr-Hole Evacuation of Chronic Subdural Haematoma: a Randomised Controlled Trial. Lancet 374 (9695), 1067–1073. 10.1016/S0140-6736(09)61115-6 19782872

[B37] Schaumann A. Klene W. Rosenstengel C. Ringel F. Tüttenberg J. Vajkoczy P. (2016). COXIBRAIN: Results of the Prospective, Randomised, Phase II/III Study for the Selective COX-2 Inhibition in Chronic Subdural Haematoma Patients. Acta Neurochir (Wien) 158 (11), 2039–2044. 10.1007/s00701-016-2949-3 27605230

[B38] Seagroatt V. Stratton I. (1998). Bias in Meta-Analysis Detected by a Simple, Graphical Test. Test Had 10% False Positive Rate. BMJ 316 (7129), 470–471. PMC2665628 9492688

[B39] Shim Y. S. Park C. O. Hyun D. K. Park H. C. Yoon S. H. (2007). What Are the Causative Factors for a Slow, Progressive Enlargement of a Chronic Subdural Hematoma? Yonsei Med. J. 48 (2), 210–217. 10.3349/ymj.2007.48.2.210 17461518 PMC2628130

[B40] Shofty B. Grossman R. (2016). Treatment Options for Chronic Subdural Hematoma. World Neurosurg. 87, 529–530. 10.1016/j.wneu.2015.09.035 26407927

[B41] Shrestha D. B. Budhathoki P. Sedhai Y. R. Jain S. Karki P. Jha P. (2021). Steroid in Chronic Subdural Hematoma: An Updated Systematic Review and Meta-Analysis Post DEX-CSDH Trial. World Neurosurg. 158, 84–99. 10.1016/j.wneu.2021.10.167 34728401

[B42] Soleman J. Lutz K. Schaedelin S. Kamenova M. Guzman R. Mariani L. (2019). In Reply: Subperiosteal vs. Subdural Drain after Burr-Hole Drainage of Chronic Subdural Hematoma: A Randomized Clinical Trial (cSDH-Drain-Trial). Neurosurgery 85 (5), E797–e34. 10.1093/neuros/nyz291 31194877

[B43] Soleman J. Nocera F. Mariani L. (2017). The Conservative and Pharmacological Management of Chronic Subdural Haematoma. Swiss Med. Wkly 147, w14398. 10.4414/smw.2017.14398 28102879

[B44] Stang A. (2010). Critical Evaluation of the Newcastle-Ottawa Scale for the Assessment of the Quality of Nonrandomized Studies in Meta-Analyses. Eur. J. Epidemiol. 25 (9), 603–605. 10.1007/s10654-010-9491-z 20652370

[B45] Stary J. M. Hutchins L. Vega R. A. (2016). Tranexamic Acid for Recurring Subdural Hematomas Following Surgical Evacuation: A Clinical Case Series. J. Neurol. Surg. A. Cent. Eur. Neurosurg. 77 (5), 422–426. 10.1055/s-0036-1584212 27300772

[B46] Sun T. F. Boet R. Poon W. S. (2005). Non-surgical Primary Treatment of Chronic Subdural Haematoma: Preliminary Results of Using Dexamethasone. Br. J. Neurosurg. 19 (4), 327–333. 10.1080/02688690500305332 16455539

[B47] Tang R. Shi J. Li X. Zou Y. Wang L. Chen Y. (2018). Effects of Atorvastatin on Surgical Treatments of Chronic Subdural Hematoma. World Neurosurg. 117, e425–e429. 10.1016/j.wneu.2018.06.047 29920396

[B48] Tanweer O. Frisoli F. A. Bravate C. Harrison G. Pacione D. Kondziolka D. (2016). Tranexamic Acid for Treatment of Residual Subdural Hematoma after Bedside Twist-Drill Evacuation. World Neurosurg. 91, 29–33. 10.1016/j.wneu.2016.03.062 27032521

[B49] Tariq J. Bhatti S. N. (2021). Adjunctive Postoperative Course of Dexamethasone in Chronic Subdural Hematoma: Effect on Surgical Outcome. Pak J. Med. Sci. 37 (7), 1877–1882. 10.12669/pjms.37.7.3374 34912411 PMC8613023

[B50] Thomas P. A. W. Marshman L. A. G. Rudd D. Moffat C. Mitchell P. S. (2019). Growth and Resorption of Chronic Subdural Hematomas: Gardner, Weir, and the Osmotic Hypothesis Revisited. World Neurosurg. 132, e202–e7. 10.1016/j.wneu.2019.08.204 31493614

[B51] van Valkenhoef G. Dias S. Ades A. E. Welton N. J. (2016). Automated Generation of Node-Splitting Models for Assessment of Inconsistency in Network Meta-Analysis. Res. Synth. Methods 7 (1), 80–93. 10.1002/jrsm.1167 26461181 PMC5057346

[B52] Wan K. R. Qiu L. Saffari S. E. Khong W. X. L. Ong J. C. L. See A. A. (2020). An Open Label Randomized Trial to Assess the Efficacy of Tranexamic Acid in Reducing post-operative Recurrence of Chronic Subdural Haemorrhage. J. Clin. Neurosci. 82 (Pt), 147–154. 10.1016/j.jocn.2020.10.053 33317724

[B53] Wang D. Gao C. Xu X. Chen T. Tian Y. Wei H. (2020). Treatment of Chronic Subdural Hematoma with Atorvastatin Combined with Low-Dose Dexamethasone: Phase II Randomized Proof-Of-Concept Clinical Trial. J. Neurosurg., 235. 10.3171/2019.11.JNS192020 32005012

[B54] Wang D. Li T. Tian Y. Wang S. Jin C. Wei H. (2014). Effects of Atorvastatin on Chronic Subdural Hematoma: a Preliminary Report from Three Medical Centers. J. Neurol. Sci. 336 (1-2), 237–242. 10.1016/j.jns.2013.11.005 24269089

[B55] Wang X. Song J. He Q. You C. (2021). Pharmacological Treatment in the Management of Chronic Subdural Hematoma. Front. Aging Neurosci. 13, 684501. 10.3389/fnagi.2021.684501 34276343 PMC8280518

[B56] Yamada T. Natori Y. (2020). Prospective Study on the Efficacy of Orally Administered Tranexamic Acid and Goreisan for the Prevention of Recurrence after Chronic Subdural Hematoma Burr Hole Surgery. World Neurosurg. 134, e549–e53. 10.1016/j.wneu.2019.10.134 31678452

[B57] Yang W. Huang J. (2017). Chronic Subdural Hematoma: Epidemiology and Natural History. Neurosurg. Clin. N. Am. 28 (2), 205–210. 10.1016/j.nec.2016.11.002 28325454

[B58] Zhang J. (2021). Expert Consensus on Drug Treatment of Chronic Subdural Hematoma. Chin. Neurosurg. J. 7 (1), 47. 10.1186/s41016-021-00263-z 34809712 PMC8607705

