# Supplementary material for: Effectiveness Comparisons of Drug Therapy on Chronic Subdural Hematoma Recurrence: A Bayesian Network Meta-Analysis and Systematic Review
Source: Front Pharmacol. 2022 Mar 17;13:845386. doi: 10.3389/fphar.2022.845386 (PMC8993499; doi:10.3389/fphar.2022.845386)
Supplement: Supplementary file 2 [file Table2.DOCX]

CSDH Chronic subdural hematoma

MeSH Medical Subject Headings

CrI Credible Interval

NMA Network Meta-analysis

SNT Standard Neurosurgical Treatment

ROB Risk of Bias

NMA Network Meta-analysis

OR Odds Ratio

RCTs Randomized Controlled Trials

SUCRA Surface Under the Cumulative Ranking curve

MCMC Markov Chain-Monte Carlo

DIC Deviance Information Criterion

PLB Placebo

TXA Tranexamic Acid

DXM Dexamethasone

ATO Atorvastatin

GRS Goreisan

CLX Celecoxib

ATB Antithrombotic
