# Supplementary material for: Effectiveness Comparisons of Drug Therapy on Chronic Subdural Hematoma Recurrence: A Bayesian Network Meta-Analysis and Systematic Review
Source: Front Pharmacol. 2022 Mar 17;13:845386. doi: 10.3389/fphar.2022.845386 (PMC8993499; doi:10.3389/fphar.2022.845386)
Supplement: Supplementary file 3 [file Table3.DOCX]

# Binomial likelihood, logit link

# Random effects model for multi-arm trials

model{ # *** PROGRAM STARTS

for(i in 1:ns){ # LOOP THROUGH STUDIES

w[i,1] <- 0 # adjustment for multi-arm trials is zero for control arm

delta[i,1] <- 0 # treatment effect is zero for control arm

mu[i] ~ dnorm(0,.0001) # vague priors for all trial baselines

for (k in 1:na[i]) { # LOOP THROUGH ARMS

r[i,k] ~ dbin(p[i,k],n[i,k]) # binomial likelihood

logit(p[i,k]) <- mu[i] + delta[i,k] # model for linear predictor

rhat[i,k] <- p[i,k] * n[i,k] # expected value of the numerators

dev[i,k] <- 2 * (r[i,k] * (log(r[i,k])-log(rhat[i,k])) #Deviance contribution

+ (n[i,k]-r[i,k]) * (log(n[i,k]-r[i,k]) - log(n[i,k]-rhat[i,k])))

}

resdev[i] <- sum(dev[i,1:na[i]]) # summed residual deviance contribution for this trial

for (k in 2:na[i]) { # LOOP THROUGH ARMS

delta[i,k] ~ dnorm(md[i,k],taud[i,k]) # trial-specific LOR distributions

md[i,k] <- d[t[i,k]] - d[t[i,1]] + sw[i,k] # mean of LOR distributions (with multi-arm trial correction)

taud[i,k] <- tau *2*(k-1)/k # precision of LOR distributions (with multi-arm trial correction)

w[i,k] <- (delta[i,k] - d[t[i,k]] + d[t[i,1]]) # adjustment for multi-arm RCTs

sw[i,k] <- sum(w[i,1:k-1])/(k-1) # cumulative adjustment for multi-arm trials

}

}

totresdev <- sum(resdev[]) #Total Residual Deviance

d[1] <- 0 # treatment effect is zero for reference treatment

for (k in 2:nt){ d[k] ~ dnorm(0,.0001) } # vague priors for treatment effects

sd ~ dunif(0,5) # vague prior for between-trial SD.

tau <- pow(sd,-2) # between-trial precision = (1/between-trial variance)

# pairwise ORs and LORs for all possible pair-wise comparisons, if nt>2

for (c in 1:(nt-1)) {

for (k in (c+1):nt) {

or[c,k] <- exp(d[k] - d[c])

lor[c,k] <- (d[k]-d[c])

}

}

# Ranking and probabilities for treatment

for(k in 1:nt) {

order[k]<- rank(d[],k) #events are bad

#order[k]<- nt+1-rank(d[],k) #events are good

most.effective[k]<-equals(order[k],1)

for(j in 1:nt) {

effectiveness[k,j]<- equals(order[k],j)

cumeffectiveness[k,j]<- sum(effectiveness[k,1:j])

}

}

#SUCRA

for(k in 1:nt) {

SUCRA[k]<- sum(cumeffectiveness[k,1:(nt-1)]) /(nt-1)

}

}

list(ns=14, nt=8) # tolerability

t[,1] t[,2] t[,3] r[,1] n[,1] r[,2] n[,2] r[,3] n[,3] na[]

1 2 NA 5 49 2 41 NA NA 2

1 4 NA 23 98 11 98 NA NA 2

1 3 NA 25 350 6 349 NA NA 2

1 3 NA 3 10 1 10 NA NA 2

1 3 NA 5 24 0 23 NA NA 2

1 3 NA 4 17 4 95 NA NA 2

1 5 NA 11 88 9 92 NA NA 2

1 2 5 8 82 1 72 7 78 3

1 6 NA 1 13 1 10 NA NA 2

1 5 NA 13 104 6 104 NA NA 2

4 7 NA 4 30 1 30 NA NA 2

1 8 NA 47 523 11 161 NA NA 2

1 3 NA 2 46 1 46 NA NA 2

1 8 NA 43 436 33 328 NA NA 2

END

#chain 1

list(d=c(NA,0,0,0,0,0,0,0), sd=1, mu=c(0,0,0,0,0,0,0,0,0,0,0,0,0,0))

#chain 2

list(d=c( NA,-1,-1,-1,-1,-1,-1,-1), sd=2, mu=c(-3,-3,-3,-3,-3,-3,-3,-3,-3,-3,-3,-3,-3,-3))

#chain 3

list(d=c( NA,2,2,2,2,2,2,2), sd=4, mu=c(5,3,2,4,-5,-4,-2,-6,2,4,1,7,-1,-5))
