# Supplementary material for: Effectiveness Comparisons of Drug Therapy on Chronic Subdural Hematoma Recurrence: A Bayesian Network Meta-Analysis and Systematic Review
Source: Front Pharmacol. 2022 Mar 17;13:845386. doi: 10.3389/fphar.2022.845386 (PMC8993499; doi:10.3389/fphar.2022.845386)
Supplement: Supplementary file 4 [file Table4.DOCX]

**1.PubMed search strategies:**

(((((((((((((((((("Tranexamic Acid"[Mesh]) OR (AMCHA[Title/Abstract])) OR (trans-4-(Aminomethyl)cyclohexanecarboxylic Acid[Title/Abstract])) OR (t-AMCHA[Title/Abstract])) OR (AMCA[Title/Abstract])) OR (Anvitoff[Title/Abstract])) OR (Cyklokapron[Title/Abstract])) OR (Ugurol[Title/Abstract])) OR (KABI 2161[Title/Abstract])) OR (Spotof[Title/Abstract])) OR (Transamin[Title/Abstract])) OR (Amchafibrin[Title/Abstract])) OR (Exacyl[Title/Abstract])) OR (((((((((((((("Dexamethasone"[Mesh]) OR (Methylfluorprednisolone[Title/Abstract])) OR (Hexadecadrol[Title/Abstract])) OR (Decameth[Title/Abstract])) OR (Decaspray[Title/Abstract])) OR (Dexasone[Title/Abstract])) OR (Dexpak[Title/Abstract])) OR (Maxidex[Title/Abstract])) OR (Millicorten[Title/Abstract])) OR (Oradexon[Title/Abstract])) OR (Decaject[Title/Abstract])) OR (Decaject-L.A.[Title/Abstract])) OR (Decaject L.A.[Title/Abstract])) OR (Hexadrol[Title/Abstract]))) OR (((((((((("Atorvastatin"[Mesh]) OR ((3R,5R)-7-(2-(4-Fluorophenyl)-5-isopropyl-3-phenyl-4-(phenylcarbamoyl)-1H-pyrrol-1-yl)-3,5-dihydroxyheptanoic acid[Title/Abstract])) OR (Atorvastatin Calcium[Title/Abstract])) OR (Atorvastatin, Calcium Salt[Title/Abstract])) OR (Liptonorm[Title/Abstract])) OR (Lipitor[Title/Abstract])) OR (Atorvastatin Calcium Hydrate[Title/Abstract])) OR (Atorvastatin Calcium Anhydrous[Title/Abstract])) OR (CI 981[Title/Abstract])) OR (Atorvastatin Calcium Trihydrate[Title/Abstract]))) OR ((((("Celecoxib"[Mesh]) OR (4-(5-(4-methylphenyl)-3-(trifluoromethyl)-1H-pyrazol-1-yl)benzenesulfonamide Celebrex[Title/Abstract])) OR (SC 58635[Title/Abstract])) OR (SC-58635[Title/Abstract])) OR (SC58635[Title/Abstract]))) OR ("oryeongsan" [Supplementary Concept])) OR (antithrombotic[Title/Abstract])) AND ((((((((("Hematoma, Subdural, Chronic"[Mesh]) OR (Subdural Hematoma, Chronic[Title/Abstract])) OR (Chronic Subdural Hematoma[Title/Abstract])) OR (Chronic Subdural Hematomas[Title/Abstract])) OR (Hematoma, Chronic Subdural[Title/Abstract])) OR (Hematomas, Chronic Subdural[Title/Abstract])) OR (Subdural Hematomas, Chronic[Title/Abstract])) OR (Hemorrhage, Subdural, Chronic[Title/Abstract]))) AND (clinical trial[Filter])

The final searches citation of the PubMed database in our NMA are as follows: <https://pubmed.ncbi.nlm.nih.gov/?term=longquery89138da57590b31e0966&filter=dates.1000%2F1%2F1-2021%2F11%2F30&timeline=expanded&page>.

**2.Embase search strategies:**

#1 'dexamethasone'/exp OR 'tranexamic acid'/exp OR 'atorvastatin'/exp OR 'goreisan'/exp OR 'celecoxib'/exp OR 'anticoagulant agent'/exp OR 'amcha':ab,ti OR 'trans-4-(aminomethyl)cyclohexanecarboxylic acid t-amcha':ab,ti OR 'amca':ab,ti OR 'anvitoff':ab,ti OR 'cyklokapron':ab,ti OR 'ugurol':ab,ti OR 'kabi 2161':ab,ti OR 'spotof':ab,ti OR 'transamin':ab,ti OR 'amchafibrin':ab,ti OR 'exacyl':ab,ti OR 'methylfluorprednisolone':ab,ti OR 'hexadecadrol':ab,ti OR 'decameth':ab,ti OR 'decaspray':ab,ti OR 'dexasone':ab,ti OR 'dexpak':ab,ti OR 'maxidex':ab,ti OR 'millicorten':ab,ti OR 'oradexon':ab,ti OR 'ha 1077':ab,ti OR 'at 877':ab,ti OR 'cardene':ab,ti OR 'vasonase':ab,ti OR 'decaject':ab,ti OR 'decaject-l.a.':ab,ti OR 'decaject l.a.':ab,ti OR 'hexadrol':ab,ti OR '(3r,5r)-7-(2-(4-fluorophenyl)-5-isopropyl-3-phenyl-4-(phenylcarbamoyl)-1h-pyrrol-1-yl)-3,5-dihydroxyheptanoic acid':ab,ti OR 'atorvastatin calcium':ab,ti OR 'atorvastatin, calcium salt':ab,ti OR 'liptonorm':ab,ti OR 'lipitor':ab,ti OR 'atorvastatin calcium hydrate':ab,ti OR 'atorvastatin calcium anhydrous':ab,ti OR 'ci 981':ab,ti OR 'atorvastatin calcium trihydrate':ab,ti OR '4-(5-(4-methylphenyl)-3-(trifluoromethyl)-1h-pyrazol-1-yl)benzenesulfonamide':ab,ti OR 'celebrex':ab,ti OR 'sc 58635':ab,ti OR 'oryeongsan':ab,ti OR 'antithrombotic':ab,ti

#2'subdural hematoma'/exp OR 'Subdural Hematoma, Chronic':ab,ti OR 'Chronic Subdural Hematoma':ab,ti OR 'Chronic Subdural Hematomas':ab,ti OR 'Hematoma, Chronic Subdural':ab,ti OR 'Hematomas, Chronic Subdural':ab,ti OR 'Subdural Hematomas, Chronic':ab,ti OR 'Hemorrhage, Subdural, Chronic':ab,ti

#3 'clinical trial'/de

#4 #1 AND #2 AND #3

The final searches citation of the Embase database in our NMA are as follows: [https://www.embase.com/?phase=continueToApp#advancedSearch/resultspage/history.6/page.1/25.items/orderby.date/source.](https://www.embase.com/?phase=continueToApp%23advancedSearch/resultspage/history.6/page.1/25.items/orderby.date/source.)

**3.Cochrane search strategies:**

#1 MeSH descriptor: [Tranexamic Acid] explode all trees OR MeSH descriptor: [Dexamethasone] explode all trees OR MeSH descriptor: [Atorvastatin] explode all trees OR MeSH descriptor: [Celecoxib] explode all trees OR MeSH descriptor: [Fibrinolytic Agents] explode all trees

#2 (AMCHA) OR (trans4cyclohexanecarboxylic Acid) OR (t-AMCHA) OR (AMCA) OR (Anvitoff) OR (Cyklokapron) OR (Ugurol) OR (KABI2161) OR (Spotof) OR (Transamin) OR (Amchafibrin) OR (Exacyl) OR (Methylfluorprednisolone) OR (Hexadecadrol) OR (Decameth) OR (Decaspray) OR (Dexasone) OR (Dexpak) OR (Maxidex) OR (Millicorten) OR (Oradexon) OR (Decaject) OR (Decaject-L.A.) OR (Decaject L.A.) OR (Hexadrol) OR (Atorvastatin Calcium) OR (Atorvastatin, Calcium Salt) OR (Liptonorm) OR (Lipitor) OR (Atorvastatin Calcium Hydrate) OR (Atorvastatin Calcium Anhydrous) OR (CI 981) OR (Atorvastatin Calcium Trihydrate) OR (Celebrex) OR (SC 58635) OR (oryeongsan) OR (antithrombotic)

#3 MeSH descriptor: [Hematoma, Subdural, Chronic] explode all trees

#4 (Subdural Hematoma, Chronic) OR (Chronic Subdural Hematoma) OR (Chronic Subdural Hematomas) OR (Hematoma, Chronic Subdural) OR (Hematomas, Chronic Subdural) OR (Subdural Hematomas, Chronic) OR (Hemorrhage, Subdural, Chronic)

#5 #1 OR #2

#6 #3 OR #4

#7 #5 AND #6

The final searches citation of the Cochrane Library database in our NMA are as follows: <https://www-cochranelibrary-com.skh.80599.net/web/cochrane/advanced-search/search-manager>
